# Factors Responsible for Gaseous Gangrene

**Published:** 1917-12

**Authors:** 


***Factors Responsible for Gaseous Gangrene.*** By KENNETH TAYLOR, Pathologist, the American Ambulance, Neuilly. Abs. from the *Lancet*, Jan. 15, 1916.

T. gives the results of experiments with the B. aerogenes capsulatus of Welch, dealing especially with the products of the bacillus responsible for the pathological changes observed in the clinical infections and in experiments upon laboratory animals.

He considers : (1) The endo-toxin contained in the bacillus; (2) The exo-toxin elaborated by the bacillus; (3) the toxin elaborated from the tissue undergoing, or which has undergone, degenerative changes due to the action of the bacillus (this agent, the author terms " tissue toxin "); and (4) the gas produced by the bacillus.

*Endo-Toxin.* — Intravenous injection of young rabbits with a dosage of 1000 million washed and killed bacilli, of a highly virulent strain per 100 grams of rabbit, and intraperitoneal injection of a 150-gram guinea pig with a dosage of 8000 million of the same bacilli produced no discernible toxic effect.

The author concludes that probably no " endo-toxin " of appreciable activity was contained in the bacillus.

*Exo-Toxin.* — The author describes his experiments as follows :

" Filtrates from sugar-free 48-hours broth cultures of three different strains of the B. aerogenes capsulatus were used. Guinea-pigs alone were employed for these experiments, and the injections of the exo-toxin made subcutaneously or intraperitoneally. For a pig of about 250 gr. the lethal dose was found, in the case of each strain, to be in the neighborhood of 10 cc. of this filtrate. Six animals receiving lethal doses died within 24 hours. Smaller doses, of from 4 to 8 cc., produced symptoms of intoxication, such as loss of power in the hind legs, rapid respiration, and refusal to eat. All the animals dying from the injections were examined post mortem. The characteristic picture was similar to that seen in the case of the actual infection. "

Further controlled experiments with fresh sterile tissue reproduced *in vitro* the same pathological changes as those seen in specimens of muscles and liver from clinical cases or experimental infection in animals.

" After 24 hours' exposure to the action of the exo-toxin, both the specimens of liver and of muscle showed on microscopical examination a much greater degree of degeneration (characterized by granular desintegration and solution of the cytoplasm of the parenchyma) than the specimen in the simple broth. After forty hours a still more marked difference was evident between the specimen treated with exo-toxin and that treated with ordinary broth. It was evident that a definite proteolytic substance was present in the exo-toxin ".

The author concludes : " The exo-toxin is of a fairly definite standard, although large doses are required to produce symptoms of intoxication. It is believed, however, that it must be considered an important part of the infection ".

*Tissue-Toxin.* — The effects of the exo-toxin upon the living animal did not appear to the author sufficient to explain the extreme degree of intoxication seen in clinical cases of gaseous gangrene. It also appears that the terminal phase of fatal cases is not due to the invasion of the blood by the bacilli, because they are rarely found in the blood before death. " It seems probable that the toxemia is due, at least in part, to the absorbtion of the autolytic products of protein digestion from the enormous amount of tissue which has been killed by the combined action of the exo-toxin and the gas. This theoretical factor is referred to as " tissue-toxin " and is perhaps a much more active factor in the intoxication than the toxin formed by the bacilli ".

*The Importance of the Gas.* — The part played by the gas was studied : (1) for its toxicity; (2) for its mechanical action.

The subcutaneous injection of 12-15 cc. of the gas after it had been freed of the carbon dioxide which constituted 30-40 per cent of the volume failed to produce any toxic effect in guinea pigs.

A case of hemothorax infected by a pure culture of the gas bacillus and showing considerable gas production, without marked toxic symptoms is also quoted as evidence of the non-toxicity of the gas.

The Author concludes : " The gas produced by this bacillus in the muscle media or in media containing large amounts of blood is of little or no importance as a toxic factor. "

In testing the *mechanical action* of the gas produced, the author performed experiments, which he describes as follows :

" Four guinea pigs of equal size were inoculated in the muscle of the thigh with one drop of a twenty-four hours' broth culture of the gas bacillus. The animals were killed at intervals of one, two, three and four hours after inoculation, the inoculated legs hardened *in toto*, cut transversely and examined. No macroscopical or microscopical change could be determined in the specimen from the animal killed one hour after inoculation. In the specimen from the animal killed two hours after inoculation, definite distension of the muscle sheaths of the inoculated thigh was evident on dissection. On microscopical examination of the hardened specimen, an extensive diffuse affection of the muscle tissues of almost the entire thigh could be readily demonstrated. The individual muscle fibres showed the characteristic separation and beginning fragmentation, evidently the result of diffusion of gas under pressure. The bacteria still remained at the site of inoculation, while the muscular degeneration extended throughout almost the entire thigh. The specimens from the animals killed three hours and four hours after inoculation showed more extensive changes of a similar character. The bacteria still seemed to be fairly well localized at the spot where they were injected.

" In order to determine the amount of pressure which might be generated in a short time by the activity of the bacillus, the indicator of an autoclave was attached by a piece of heavy rubber tubing to a glass tube containing 10 cc. of dextrose broth culture of the bacillus, and the apparatus incubated. Pressure began to rise about two hours after inoculation, and after five hours it had reached 1 1/2 atmospheres (23 pounds) of pressure when the rubber connexion blew off the indicator. "

From these experiments the author concludes : " The actively growing bacilli are capable of producing a pressure which must be extremely destructive to living tissue, and, further, that this pressure must be generated within a very short period of time if the process is, as frequently occurs, restrained by a firm muscle sheath. This pressure must, of course, produce a complete anemia of the muscle within which the pressure is generated and maintain this anemia until the rupture of the muscle sheath occurs. In addition to bringing about the death of the tissue by anemia, it is apparent from microscopical examination of muscles which have been subjected to these high pressures that there is an actual fragmentation of the individual fibers with rupture of the fiber sheaths and disintegration of the fiber substance. "

The author concludes further that the subcutaneous crepitation which is so often a symptom, is probably produced by gas which has forced itself through the muscle sheaths, and not by gas produced by the bacillus in this tissue, where, indeed, it is rarely to be found. He believes that when such passage is occluded, the explosive type of the infection may be the result. He thinks it also probable that the bacteria are scattered by the gas pressure, especially when it causes the rupture of restraining tissues.

T. calls attention to the new surgical problem involved in the effective drainage of the gas instead of a purulent material. The bulging of the muscle often closes surgical incisions. Incisions parallel to the muscles are inadequate. Transverse section is impraticable. The author believes that the success of many amputations in serious cases is in part due to the opportunity for effective drainage made possible by the transverse section.

The author further concludes :

" 1. The gas produced by the B. aerogenes capsulatus is of little or no importance as a toxic factor.

" 2. The mechanical action of the pressure produced is usually, if not always, the most important part of the infection. To it may be charged the development of highly pathogenic possibilities in a usually rather innocent infection. It brings about (*a*) the death of the tissues from the resulting anemia produced by a pressure much higher than that of the circulating blood; (*b*) the actual mechanical fragmentation of the tissues, especially muscle; and (*c*) the mechanical scattering of the infection.

" 3. One of the chief problems in the treatment of the infection is that of establishing drainage for the escape of the gas before the pressure has resulted in the death of the tissue. "

